# Serum Procalcitonin as a Predictive Biomarker in COVID-19: A Retrospective Cohort Analysis

**DOI:** 10.7759/cureus.27816

**Published:** 2022-08-09

**Authors:** Aaiz Hussain, Lavi Singh, James McAlister III, Yongho Jo, Tadevos T Makaryan, Shaheer Hussain, Robert W Trenschel, Marc M Kesselman

**Affiliations:** 1 College of Medicine, Nova Southeastern University, Dr. Kiran C. Patel College of Allopathic Medicine, Davie, USA; 2 College of Medicine, Wayne State University, Detroit, USA; 3 Public Health, Nova Southeastern University, Dr. Kiran C. Patel College of Allopathic Medicine, Davie, USA; 4 College of Medicine, Chicago College of Osteopathic Medicine, Midwestern University, Downers Grove, USA; 5 Rheumatology, Nova Southeastern University, Dr. Kiran C. Patel College of Osteopathic Medicine, Davie, USA

**Keywords:** infection, respiratory viral infect, serum biomarkers, covid 19, procalcitonin

## Abstract

Introduction: Since the onset of COVID-19, physicians and scientists have been working to further understand biomarkers associated with the infection, so that patients who have contracted the virus can be treated. Although COVID-19 is a complex virus that affects patients differently, current research suggests that COVID-19 infections have been associated with increased procalcitonin, a biomarker traditionally indicative of bacterial infections. This paper aims to investigate the relationship between COVID-19 infection severity and procalcitonin levels in the hopes to aid the management of patients with COVID-19 infections.

Methods: Patient data were obtained from the Renaissance School of Medicine at Stony Brook University. The data of the patients who had tested positive for COVID-19 and had an associated procalcitonin value (n=1046) was divided into age splits of 18-59, 59-74, and 74-90. Multiple factors were analyzed to determine the severity of each patient’s infection. Patients were divided into low, medium, and high severity dependent on the patient's COVID-19 severity. A one-way analysis of variance (ANOVA) was done for each age split to compare procalcitonin values of the severity groups within the respective age split. Next, post hoc analysis was done for the severity groups in each age split to further compare the groups against each other.

Results: One-way ANOVA testing of the three age splits all had a resulting p<0.0001, displaying that the null hypothesis was rejected. In the post hoc analysis, however, the test failed to reject the null hypothesis when comparing the medium and high severity groups against each other in the 59-74 and 74-90 age splits. The null hypothesis was rejected in all pairwise comparisons in the 18-59 age split. We determined that a procalcitonin value of greater than 0.24 ng/mL would be characterized as a more severe COVID-19 infection when considering patient factors and comorbidities.

Conclusion: The analysis of the data concluded that elevated procalcitonin levels correlated with the severity of COVID-19 infections. This finding can be used to assist medical providers in the management of COVID-19 patients.

## Introduction

The emergence of biomarkers as a diagnostic tool has afforded clinicians an objective method to assess disease presence, severity, and other useful clinical information. With the ongoing COVID-19 pandemic, there has been an effort to identify biomarkers with which to stratify patients. These biomarkers range from basic hematologic parameters to cytokines and acute phase reactants. Current research efforts have sought to use the rise and fall of certain biomarkers to identify where patients lie in the course of the infection and predict disease severity and diagnostic outcomes [1].

The pathogenesis of a COVID-19 infection is complex and variable amongst individuals and differs over multiple parameters such as age, gender, and comorbidities. Current research shows an interaction between the viral (Sars-Cov-2) spike protein S and Angiotensin Converting Enzyme 2 (ACE2) receptor as a method of entry into a cell [2]. Not only is this a method of entry, but the virus’s interactions with the ACE2 receptor lead to an imbalance between the pro-inflammatory and anti-inflammatory effects of Angiotensin II on the Angiotensin I and Angiotensin II receptors [3]. Once inside, the virus initiates an innate and adaptive immune response which leads to several changes such as the release of several cytokines and chemokines and causes other immune changes such as lymphopenia and neutrophilia [4]. The massive surge of cytokines that results from viral invasion and cellular attachment is distributed around the body, further exacerbating systemic inflammation [3]. During this process, there are marked variations in levels of inflammatory biomarkers, such as an increase in Interleukin-6 (IL-6), Lactate dehydrogenase (LDH), C-reactive protein (CRP), D-Dimer, and Ferritin levels. These inflammatory markers are being utilized to predict the severity of illness, disease course, and response to treatment [5-9]. Furthermore, Yitbarek et al. found that CRP is a biomarker that can predict the severity of COVID-19 disease [10].

A particularly interesting and potentially significant biomarker is procalcitonin. This biomarker is an acute phase reactant and has traditionally been associated with bacterial infections is significantly elevated in bacterial septic patients [11]. However, changes in procalcitonin levels have also been seen in patients with severe COVID-19 infections [12]. The suspected mechanism relates to the production of lipopolysaccharide by bacteria as well as the release of bacteria-specific cytokines. The strength of the association between procalcitonin and sepsis has made it a tool to guide empiric antibiotic usage and promote antibiotic stewardship [11]. A prospective study that looked at patients who presented to an ED with suspected infection found a specificity of 0.99 and sensitivity of 0.35 when using a cutoff point for procalcitonin of 0.05 ng/ml to determine the diagnosis of systemic infection [13]. In contrast, during viral infections, procalcitonin is thought to be suppressed by interferon signaling, which is elevated in viral infections. Gautam et al. found that patients with a viral infection and a bacterial coinfection had higher procalcitonin values than those with pure viral infections (p < 0.001). However, the study also found that when the pure viral infections were severe enough, procalcitonin rose. This phenomenon was attributed to the severity of the viral infectious process overall, causing a rise in procalcitonin levels regardless of interferon inhibition [14].

Thus, it has been observed that levels of procalcitonin may rise in patients with severe COVID-19 sepsis. Lippi et al., in their meta-analysis, found a fivefold increase in procalcitonin values in predicting severe COVID-19 infection but commented on the limited data available, and thus only suggesting a trend may exist [12]. Proposed explanations for this include superimposed bacterial superinfection; however, the exact reason for this finding has yet to be fully explained [15].

In an effort to further explore the prognostic value of this biomarker, we sought to use publicly available datasets to correlate COVID-19 severity to procalcitonin levels. Strengthening the association between COVID-19 severity and levels of procalcitonin could aid in the management of COVID-19 patients. For example, it could serve as an additional metric in current severity algorithms allowing for more accurate prognostic predictions and as a harbinger of disease severity, prompting more aggressive treatment.

## Materials and methods

Data source and variables

We accessed a public dataset provided to NIH’s database: Open-Access Data and Computational Resources to Address COVID-19. The dataset was collected by and provided in August of 2021 by the Renaissance School of Medicine at Stony Brook University [16]. It contained the clinical data of 1384 patients who had tested positive for COVID-19. The dataset included imaging data, hospital stay data, lab values, and other information. To determine if procalcitonin can be correlated to the severity of COVID-19 infection, we excluded 338 of 1384, as these 338 patients did not have procalcitonin values provided. Doing so left us with 1046 patients. The 1046 patients were split into their respective age splits, which were 18 to 59, 59 to 74, and 74 to 90. We then extracted a few pieces of clinical data to determine the severity of the patients’ COVID-19 infection. These were 1) last.status: whether the patient eventually got discharged from the hospital or passed away, 2) is_icu: whether the patient was admitted to the ICU during their stay or not, 3) was.ventilated: whether the patient was mechanically ventilated during their stay or not, and 4) length_of_stay: the number of days the patient stayed in the hospital. Lastly, we extracted the respective procalcitonin (ng/mL) values for each patient.

Each of these COVID-19 severity qualifiers then received points. Each patient started with one point for testing positive for COVID-19. After that, if a patient’s last status was deceased, they received three points. If they were admitted to the ICU they received one point. If they were mechanically ventilated, they received two points, and lastly, if their length of stay was greater than 13 days, they received one point. We determined 13 days as the cutoff for the length of stay, as on average, according to the CDC, COVID-19 survivors stay in the hospital for 10-13 days [17]. Patients with 1 to 2 points were characterized as low severity, 3 to 5 were medium severity, and 6 to 8 were high severity. We also extracted the patients’ comorbidities for further analysis, which included hypertension, diabetes, coronary artery disease, heart failure, chronic kidney disease, malignancy, COPD, smoking history, or another lung disease.

Statistical analysis

Once the patients were divided into their respective age splits and further categorized into their COVID-19 severity group, statistical outliers were removed to prevent skewing of the data. In order to determine a statistical outlier, the standard IQR rule was implemented. To achieve this, Q1, Q2 (median), and Q3 were found for each category by sorting the data from lowest to highest values. Once the three quartiles were obtained, the interquartile range (IQR) was calculated, which is Q3 - Q1. With the IQR rule, all values above or below 1.5*IQR are considered statistical outliers. Once this process was repeated for all groups, the data could now be properly analyzed for further understanding. One hundred twenty-six total outliers were removed from 1046 patients to leave 920 patients remaining.

With the outliers removed, the mean was obtained for each categorization of the data. For each mean, a confidence interval was calculated using the standard method of 95% confidence, along with the standard deviations. At this point, the trend in the data appeared to support our hypothesis, as there appeared to be a positive trend with procalcitonin levels and severity. However, to determine if the data was statistically significant, we performed a one-way ANOVA test for each age split. The three groups for each age split were the respective COVID-19 severities: low, medium, and high. Next, posthoc analysis was performed to compare each severity group within each age split against another using the Tukey HSD method to assess for potential Type I errors, given the standard 95% confidence of analysis. Statistical analysis was performed using STATA 16.1 (2019, StataCorp LLC). P-values <0.05 were considered statistically significant.

Statement of ethics

The database used for this study contains de-identified patient data; therefore, no consent form or Institutional Review Board (IRB) was required to be approved.

## Results

Patient demographics

There were a total of 1046 patients who tested positive for COVID-19 and had a procalcitonin value (ng/ml). After outliers were removed, 920 patients remained. These patients were further divided into three categories corresponding to their age demographic. The age splits were 18 to 59, 59 to 74, and 74 to 90. The 18 to 59 age split had 443 patients. When the patients in this age split were further divided based on COVID-19 severity, there were 24 patients with high severity COVID-19, there were 76 patients with medium severity COVID-19, and finally 343 patients with low severity COVID-19. The 59 to 74 age split had 262 patients. When the patients in this age split were further divided based on COVID-19 severity, there were 45 patients with high severity COVID-19, there were 44 patients with medium severity COVID-19, and finally 173 patients with low severity COVID-19. The 74 to 90 age split had 215 patients. When the patients in this age split were further divided based on COVID-19 severity, there were 22 patients with high severity COVID-19, there were 61 patients with medium severity COVID-19, and finally 132 patients with low severity COVID-19.

Analysis

After performing one-way ANOVA on each age split with their respective subdivided COVID-19 severity, p-values were obtained (Tables 1-3). Results demonstrate for every age split: the p-value is <0.0001. This means a significant difference exists between the three severity groups within each age split. However, since we do not know if the difference is between all groups or just two groups, our next step was to perform post-hoc analysis pairwise comparisons.

**Table 1:** One-way ANOVA results for 18-59 age split. ANOVA: Analysis of variance, COVID-19: Coronavirus disease 2019, ME: Margin of error, N: Number of values, P-Value: Probability value

COVID-19 Severity	Mean ± 95% ME	N	Standard Deviation	Standard Error	P-Value (95% Confidence)
Low	0.1436 ± 0.00931	343	0.088	0.0047	<0.0001
Medium	0.2968 ± 0.0546	76	0.2426	0.0278	
High	1.0492 ± 0.446	24	1.1157	0.2278	

**Table 2:** One-way ANOVA results for 59-74 age split. ANOVA: Analysis of variance, COVID-19: Coronavirus disease 2019, ME: Margin of error, N: Number of values, P-Value: Probability value

COVID-19 Severity	Mean ± 95% ME	N	Standard Deviation	Standard Error	P-Value (95% Confidence)
Low	0.1688 ± 0.0171	173	0.1147	0.0087	<0.0001
Medium	0.3618 ± 0.093	44	0.3149	0.0475	
High	0.3824 ± 0.0936	45	0.3204	0.0478	

**Table 3:** One-way ANOVA results for 74-90 age split. ANOVA: Analysis of variance, COVID-19: Coronavirus disease 2019, ME: Margin of error, N: Number of values, P-Value: Probability value

COVID-19 Severity	Mean ± 95% ME	N	Standard Deviation	Standard Error	P-Value (95% Confidence)
Low	0.1477 ± 0.0153	132	0.0898	0.0078	<0.0001
Medium	0.3977 ±0.11	61	0.4383	0.0561	
High	0.3891 ±0.166	21	0.3975	0.0847	

Post-hoc analysis was done for each of the age splits using the Tukey HSD method (Tables 4-6). The post-hoc analysis more clearly demonstrates where the differences in the ANOVA test stem from. In Table 4, there is a significant difference between each severity group in the 18-59 age split (p = 0.001). This is not the case in the 59-74 age split, and the 74-90 age splits, Table 5 and Table 6, respectively. While the low to medium and low to high pairwise comparisons has p=0.001, the medium to high pairwise comparisons has p=0.875 for the 59-74 age split and p=0.9 for the 74-90 age split (Tables 5-6).

**Table 4:** Post-hoc pairwise comparisons for 18-59 age split. P-Value: Probability value

Pairwise Comparison of Severity Groups	Difference in Mean	95% Confidence Interval	P-Value (95% Confidence)
Low to Medium	0.153	0.068 to 0.238	0.001
Low to High	0.906	0.764 to 1.047	0.001
Medium to High	0.752	0.595 to 0.909	0.001

**Table 5:** Post-hoc pairwise comparisons for 59-74 age split. P-Value: Probability value

Pairwise Comparison of Severity Groups	Difference in Mean	95% Confidence Interval	P-Value (95% Confidence)
Low to Medium	0.193	0.111 to 0.275	0.001
Low to High	0.214	0.132 to 0.295	0.001
Medium to High	0.021	-0.083 to 0.124	0.875

**Table 6:** Post-hoc pairwise comparisons for 74-90 age split. P-Value: Probability value

Pairwise Comparison of Severity Groups	Difference in Mean	95% Confidence Interval	P-Value (95% Confidence)
Low to Medium	0.250	0.150 to 0.350	0.001
Low to High	0.241	0.092 to 0.390	0.001
Medium to High	-0.009	-0.169 to 0.152	0.9

The comorbidities of patients in the high severity COVID-19 category (with removed outliers) for each age split were also extracted for analysis (Tables 7-9). These are discussed further in the discussion section below.

**Table 7:** Associated comorbidities of patients with high COVID-19 severity in the 18-59 age split. COVID-19: Coronavirus disease 2019, ng: nanogram, ml: milliliters, DM: Diabetes mellitus, CKD: Chronic kidney disease, HTN: Hypertension, Other Lung Ds: Other lung disease, CAD: Coronary artery disease, COPD: Chronic obstructive pulmonary disease

COVID-19 Severity	Procalcitonin (ng/ml)	Comorbidity
8	4.02	DM, CKD
8	1.65	None
8	1.18	HTN, DM
8	0.81	HTN, Other Lung Ds
8	0.6	None
8	0.6	None
8	0.52	None
8	0.49	None
8	0.43	None
8	0.29	DM
8	0.26	HTN
8	0.26	HTN
8	0.23	None
8	0.23	Other Lung Ds, Former Smoker
8	0.17	DM
8	0.11	Other Lung Ds
8	0.09	None
7	3.44	HTN, DM, CAD
7	2.8	None
7	2.41	HTN, DM
7	1.94	Other Lung Ds
7	1.26	HTN, DM, Other Lung Ds
7	1.21	None
7	0.18	COPD, Other Lung Ds, Former Smoker

**Table 8:** Associated comorbidities of patients with high COVID-19 severity in the 59-74 age split. COVID-19: Coronavirus disease 2019, ng: nanogram, ml: milliliters, DM: Diabetes mellitus, CKD: Chronic kidney disease, Malig: Malignancy, HTN: Hypertension, Other Lung Ds: Other lung disease, CAD: Coronary artery disease, COPD: Chronic obstructive pulmonary disease, HFrEF: Heart failure with reduced ejection fraction

COVID-19 Severity Score	Procalcitonin (ng/ml)	Comorbidity
8	1.22	None
8	1.2	None
8	1.19	HTN, CAD, Malig
8	1.02	HTN, DM, CAD
8	0.89	HTN, CAD
8	0.73	DM
8	0.7	HTN, DM, Malig, Former Smoker
8	0.63	HTN, DM, CAD, Other Lung Ds, Former Smoker
8	0.49	HTN, Other Lung Ds
8	0.4	HTN
8	0.38	COPD, Former Smoker
8	0.34	HTN, DM
8	0.31	Malig, Never Smoker
8	0.31	DM, Former Smoker
8	0.29	None
8	0.29	HTN
8	0.28	HTN, Former Smoker
8	0.26	None
8	0.24	HTN, DM, CAD, Former Smoker
8	0.22	Former Smoker
8	0.22	HTN, DM
8	0.19	HTN, CAD
8	0.18	HTN
8	0.17	HTN, DM, Malig
8	0.16	HTN, CAD, HFrEF, CKD, Former Smoker
8	0.14	HTN, DM, CAD, Former Smoker
8	0.12	HTN, DM, Malig, Former Smoker
8	0.11	Former Smoker
8	0.1	HTN, DM, CAD
8	0.1	Other Lung Ds
8	0.08	HTN, DM
8	0.06	None
8	0.06	None
7	0.8	Current Smoker
7	0.65	HTN, CKD
7	0.54	HTN, DM, Former Smoker
7	0.34	HTN, DM, CAD, CKD, Other Lung Ds, Former Smoker
7	0.33	HTN, DM
7	0.29	None
7	0.26	HTN, Former Smoker
7	0.21	HTN, DM, COPD, Former Smoker
7	0.13	HTN, DM, CAD, CKD
7	0.13	HTN, CAD, COPD, Other Lung Ds, Former Smoker
7	0.04	HTN, DM, CAD, HFrEF
6	0.41	HTN

**Table 9:** Associated comorbidities of patients with high COVID-19 severity in the 74-90 age split. COVID-19: Coronavirus disease 2019, ng: nanogram, ml: milliliters, DM: Diabetes mellitus, CKD: Chronic kidney disease, Malig: Malignancy, HTN: Hypertension, Other Lung Ds: Other lung disease, CAD: Coronary artery disease, COPD: Chronic obstructive pulmonary disease

COVID-19 Severity	Procalcitonin (ng/ml)	Comorbidity
8	1.43	HTN, DM
8	1.16	HTN, Former Smoker
8	0.76	HTN
8	0.33	HTN, CAD
8	0.24	HTN, CAD, COPD, Other Lung Ds
8	0.24	HTN, DM, CAD, HFpEF, CKD, COPD, Other Lung Ds, Former Smoker
8	0.24	HTN, Former Smoker
8	0.19	HTN, Former Smoker
8	0.18	None
8	0.13	HTN, DM, CAD, HFrEF, Other Lung Ds, Former Smoker
8	0.12	HTN, CAD, Former Smoker
8	0.1	HTN, DM, CAD
8	0.1	None
7	1.2	Malig, Former Smoker
7	0.39	HTN, DM, CAD, HFpEF, CKD
7	0.28	HTN, Other Lung Ds
7	0.23	None
7	0.23	HTN, CAD, Malig
7	0.15	None
7	0.12	HTN, DM, Former Smoker
7	0.06	HTN, Malig, Former Smoker
6	0.68	None

## Discussion

As demonstrated by our results, a one-way ANOVA displayed a p-value < 0.0001 for each age split (Tables 1-3), i.e., increased procalcitonin correlated with higher COVID-19 severity in the patients. We further conducted a post-hoc analysis of the three one-way ANOVA groups (Tables 4-6). In the 18-59 age splits, each pairwise comparison had a p-value of 0.001. Thus, in the 18-59 age split, there was a significant difference in the procalcitonin values with increasing COVID-19 severity. However, in the 59-74 and 74-90 age splits, the null hypothesis was only rejected between the low severity groups when compared to the medium and high severity groups, respectively (p-value = 0.001). There was no significant difference between the medium and high severity groups in the 59-74 and 74-90 age splits (p values of 0.875 and 0.9, respectively). A cause of this discrepancy in these two older age splits may be due to either the smaller sample size in the medium and high severity groups or the arbitrary nature of how we determined the scoring for the COVID-19 severity. These two limitations are further discussed in the limitations section below. Further diving into these results, a generalized conclusion can be made that procalcitonin values increase from low severity to increased severity in every age group. The low severity category was defined as patients who tested positive for COVID-19 and were either admitted to the ICU or stayed in the hospital for more than 13 days. However, if they either needed the ventilator or needed to go to the ICU after being in the hospital for more than 13 days or stayed in the hospital for more than 13 days after being in the ICU, then their COVID-19 severity went to a higher severity group. A more useful piece of information/conclusion would be that a patient admitted to the ICU or in the hospital for more than 13 days would have a higher chance of requiring the ventilator or even dying if higher procalcitonin values are seen. To accommodate for each age split and 95% ME, a procalcitonin value of greater than 0.24 ng/mL would be characterized as a more severe COVID-19 infection. Various other studies have made conclusions similar to our studies. Carbonell et al. concluded that elevated procalcitonin level at admission was associated with higher mortality, independent of the possible bacterial co-infection [18]. Surme et al. found that high procalcitonin levels were associated with worse outcomes and higher mortality in ICU patients [19]. Tong-Minh et al. showed a higher rate of ICU admission and mortality in patients with higher procalcitonin levels in the emergency department [20]. Kaal et al. also determined that patients with procalcitonin levels above 0.1 ng/mL were at a higher risk of severe COVID-19 infection [21].

Limitations

Our study encompasses a small sample size with a total of 920 patients, which hinders the generalizability of the study as patients. The arbitrary nature of how we determined the COVID-19 severity can also be considered a major limitation of our study. However, we deemed it acceptable for the data we employed. One confounding variable that may have skewed our data was that in each group of patients, higher severity cases of COVID-19 infections were found in patients having more comorbidities. Patients in each age split with high severity COVID-19 infections, all had multiple comorbidities (Tables 7-9). This may act as a confounder because their comorbidities may result in their procalcitonin values being elevated already at baseline prior to infection due to the inflammatory nature of their comorbidities. For example, Wang et al. demonstrated that procalcitonin can be employed to predict risk of infection in T2DM patients, as it is elevated in such patients [22]. This may thus have affected the overall data in skewing procalcitonin levels to higher values. Lastly, to our knowledge the database we employed did not specify the COVID-19 variant that was studied.

## Conclusions

In conclusion, higher procalcitonin values are correlated with increased COVID-19 severity. Overall, while procalcitonin has been demonstrated to have a correlation as a predictive biomarker for bacterial sepsis and to guide antibiotic therapy, our study demonstrated a possible correlation of increasing procalcitonin values with higher COVID-19 severity. Thus, it may provide clinical value in predicting prognosis and managing care of COVID-19 patients.
